# Symptomatic cluster headache: a review of 63 cases

**DOI:** 10.1186/2193-1801-3-64

**Published:** 2014-02-03

**Authors:** Bengt Edvardsson

**Affiliations:** Department of Clinical Sciences Lund, Faculty of Medicine, Neurology, Skane University Hospital, S-221 85, Lund, Sweden

**Keywords:** Cluster headache, Neuroimaging, Secondary, Symptomatic, Magnetic resonance imaging, Differential diagnosis

## Abstract

Cluster headache is a primary headache by definition not caused by any known underlying structural pathology. Symptomatic cases have been described, for example tumours, dissections and infections, but a causal relationship between the underlying lesion and the headache is difficult to determine in many cases. The proper diagnostic evaluation of cluster headache is an issue unresolved. The literature has been reviewed for symptomatic cluster headache or cluster headache-like cases in which causality was likely. The review also attempted to identify clinical predictors of underlying lesions in order to formulate guidelines for neuroimaging. Sixty-three cluster headache or "cluster headache-like"/"cluster-like headache" cases in the literature were identified which were associated with an underlying lesion. A majority of the cases had a non-typical presentation that is atypical symptomatology and abnormal examination (including Horner’s syndrome). A striking finding in this appraisal was that a significant proportion of CH cases were secondary to diseases of the pituitary gland or pituitary region. Another notable finding was that a proportion of cluster headache cases were associated with arterial dissection. Even typical cluster headaches can be caused by structural lesions and the response to typical cluster headache treatments does not exclude a secondary form. It is difficult to draw definitive conclusions from this retrospective review of case reports especially considering the size of the material. However, based on this review, I suggest that neuroimaging, preferably contrast-enhanced magnetic resonance imaging/magnetic resonance angiography should be undertaken in patients with atypical symptomatology, late onset, abnormal examination (including Horner’s syndrome), or those resistant to the appropriate medical treatment. The decision to perform magnetic resonance imaging in cases of typical cluster headache remains a matter of medical art.

## Introduction

Cluster headache (CH) is a primary headache, by definition not caused by any underlying structural pathology and belonging to the group of trigeminal-autonomic cephalalgias (Headache Subcommittee of the International Headache Society 2004). CH is the most frequent syndrome in this group. The characteristic symptoms are strictly unilateral head pain (mainly around orbital and temporal regions) and associated ipsilateral cranial autonomic features. The headache usually lasts 45 to 90 minutes, but can range between 15 and 180 minutes. CH is an excruciating headache and probably one of the most painful headache syndromes. A circannual and circadian pattern is typical. Most patients have the episodic form characterized by bouts lasting >1 week and separated by remissions lasting longer than 4 weeks. A minority of about 10% to 20% have the chronic form, with no remission within a year or remission periods lasting <1 month (Headache Subcommittee of the International Headache Society 2004; Cittadini & Matharu 2009). The disease primarily emerges between the ages of 20 to 40 years and is more prevalent in men. Pooled data from epidemiological studies give CH a lifetime prevalence of 0.12%. Furthermore, the condition has a heritable tendency in some families (Nesbitt & Goadsby 2012). The pathophysiology of CH is not well known. The most widely accepted theory is that primary CH is characterized by hypothalamic activation with secondary activation of the trigeminal-autonomic reflex, probably by a trigeminal-hypothalamic pathway (Nesbitt & Goadsby 2012). Cluster headache has also been associated with heart disease. Data indicate a higher prevalence of right-left shunt in patients with cluster headache compared with controls (Morelli et al. 2005). The great majority of cases of CH are primary. There are many case reports of symptomatic/secondary cases of CH in the literature (Table 1), but a causal relationship between the underlying lesion and the headache is difficult to determine in many cases (Cittadini & Matharu 2009). The proper diagnostic evaluation of CH is an issue unresolved. The true prevalence of symptomatic cases of CHs is unknown because there are no prospective population-based studies including neuroradiology. Thus, the question arises whether patients with CH should have a diagnostic evaluation to rule out an underlying structural lesion. To address this question, I reviewed the English language literature on symptomatic/secondary cases of CHs. The review also attempts to identify clinical predictors of underlying lesions in order to formulate guidelines for neuroimaging.

**Table 1:** **Conditions associated with cluster headache**

•	Arterial aneurysm [West & Todman 1991, Todo & Inoya 1991, McBeath & Nanda 2000, Gentile et al. 2006, Valença et al. 2007 case 1 and 2]*
•	Arteriovenous malformation-/cavernous hemangioma [Mani & Deeter 1982, Munoz et al. 1996 case 1 and 2, Favier et al. 2007a case 3, Sewell et al. 2009
•	Subclavian steal syndrome [Piovesan et al. 2001
•	Carotid artery thrombosis [Ashkenazi & Brown 2008
•	Cerebral venous thrombosis [Park et al. 2006, Peterlin et al. 2006, Georgiadis et al. 2007, Rodríguez et al. 2008
•	Carotid-/vertebral dissection [Mainardi et al. 2002, Frigerio et al. 2003, Hannerz et al. 2005, Razvi et al. 2006, Rigamonti et al. 2007 case 1 and 2, Hardmeier et al. 2007, Straube et al. 2007 case 2, Godeiro-Junior et al. 2008, Tobin & Flitman 2008, Kim et al. 2008
•	Pituitary tumours [Tfelt-Hansen et al. 1982, Greve & Mai 1988 case 3, Milos et al. 1996, Porta-Etessam et al. 2001, Minguzzi et al. 2003, Negoro et al. 2005, Favier et al. 2007a case 2 and 4, Levy et al. 2012, Edvardsson 2013
•	Meningeoma [Kuritzky 1984, Hannerz 1989, Taub et al. 1995, Alty et al. 2008, Robbins et al. 2009
•	Glioblastoma multiforme [Edvardsson & Persson 2012
•	Hemangiopericytoma [Fontaine et al. 2013
•	Nasopharynx carcinoma [Appelbaum & Noronha 1989
•	Angiomyolipoma [Messina et al. 2013
•	Epidermoid tumour [Levyman et al. 1991, Massie et al. 2006, Eimil-Ortiz et al. 2008
•	Inflammatory myofibroblastic tumour [Bigal et al. 2003
•	Lipoma [Cologno et al. 2008
•	Arachnoid cyst [Edvardsson & Persson 2013a
•	Sinusitis [Takeshima et al. 1988, Edvardsson & Persson 2013b
•	Aspergilloma [Zanchin et al. 1995
•	Granolomatous pituitary involvement [Favier et al. 2007b
•	Orbital pseudotumour [Harley & Ahmed 2008
•	Cervical spinal epidural abscess [Liu & Su 2009
•	Multiple sclerosis [Gentile et al. 2007
•	Foreign body in the maxillary sinus [Scorticati et al. 2002
•	Cervical syringomyelia and Arnold -Chiari malformation [Seijo-Martinez et al. 2004
•	Sarcoidosis [van der Vlist et al. 2013

*References.

## Materials and methods

A literature search of English-language articles in PubMed using the keywords "cluster headache", "secondary", "symptomatic", "infection", "inflammation", "multiple sclerosis", "tumour", "vascular", "malformation", "infarction", "malignancy" was conducted. Four own published cases were also included in the review. Only articles with a diagnosis of "cluster headache" or "cluster headache-like/cluster-like headache" were included. The search has been carried out from 1993 to May 2013, but also older mentioned publications (in articles) were included. Both original articles and review articles were evaluated. The purpose of the search was to identify symptomatic headaches caused by a reported underlying lesion. Cases of headache which developed in the context of or was directly associated with trauma, stroke, and operations/interventions such as dental surgeries, neck surgery and eye surgery were excluded. Only articles with a clear description of the localization of the underlying lesion and headache were included and only articles where a therapeutic intervention directed at the underlying lesion had resulted in a significant improvement or resolution of the headache. A causal relationship in all these cases is likely but unproven.

## Results

**Figure 1:**
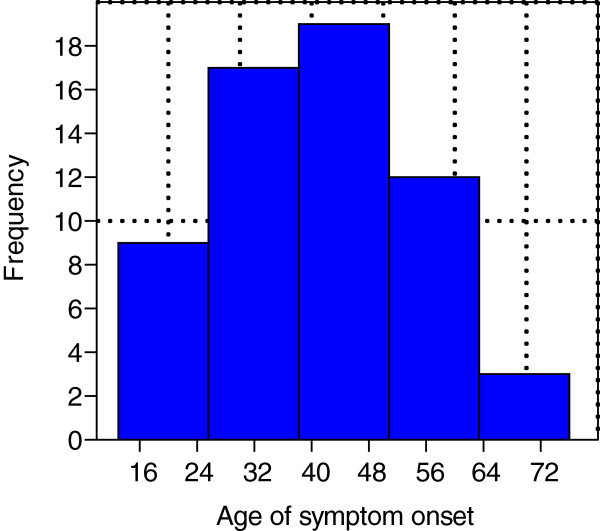
**Age of symptom onset.** Legend: Mean (± 14) age of symptom onset was 40.

**Figure 2:**
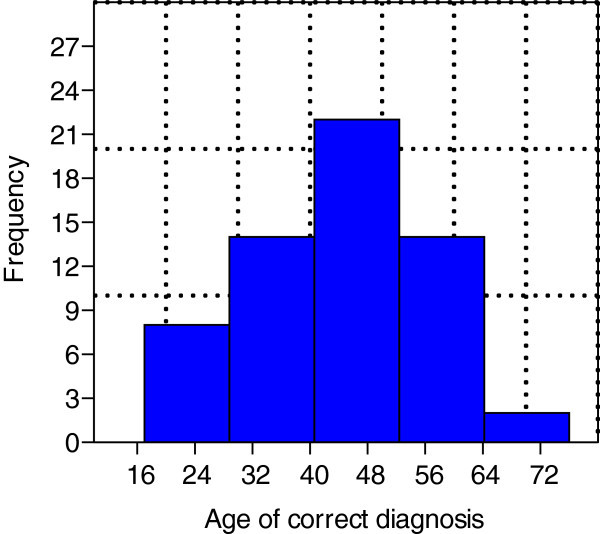
**Age of correct diagnosis.** Legend: Mean (± 13) age of correct diagnosis was 44.

The initial search identified 375 papers of cluster headache or "cluster headache-like"/"cluster-like headache". Sixty-three cluster headache or "cluster headache-like"/"cluster-like headache" cases (including 4 of my own) were found (Table 1). All cases had a clear description of the localization of the underlying lesion and were only included if a therapeutic intervention directed at the underlying lesion had resulted in a significant improvement or resolution of the headache. All other cases were excluded due to insufficient data or lack of relevance in the papers. Forty-eight (76%) of the sample were male and 15 female, the M: F ratio being 3.2:1. The mean age of symptom onset was 40 years ± 14 (range: 13–76 years) (Figure 1). The mean age of correct diagnosis was 44 years ± 13 (range: 17–76 years) (Figure 2).

In 28 patients (44%) a vascular cause was identified, including arterial aneurysms, arteriovenous malformation/cavernous angioma, venous sinus thrombosis, carotid/vertebral dissection, subclavian steal syndrome, cavernous carotid artery thrombosis, moyamoya disease, of which 11 had a dissection. Twenty-five patients (40%) had a tumour including 10 with pituitary tumours. An arachnoid cyst and a meningioma were also found in the pituitary region. Inflammation/infection accounted for 7 cases (11%), of which 1 with granulomatous hypophysitis and 1 with hypothalamic sarcoidosis. The remaining 3 patients had multiple sclerosis, foreign body and Arnold-Chiari malformation with cervical syringomyelia.

Thirty patients (48%) satisfied the criteria for CH (Headache Subcommittee of the International Headache Society 2004). The remaining 33 patients had an atypical presentation (Table 2).

**Table 2:** **Atypical presentation/atypical symptoms associated with cluster headache**

•	Atypical attacks duration [Mani & Deeter 1982, West & Todman 1991, Todo & Inoya 1991, Zanchin et al. 1995, Milos et al. 1996, Mainardi et al. 2002, Razvi et al. 2006, Park et al. 2006, Massie et al. 2006, Favier et al. 2007a case 2, Rigamonti et al. 2007 case 1, Hardmeier et al. 2007, Cologno et al. 2008, Tobin & Flitman 2008, Eimil-Ortiz et al. 2008, Robbins et al. 2009, Liu & Su 2009*
•	Atypical attack frequency [Todo & Inoya 1991, Favier et al. 2007a case 2 and 3, Alty et al. 2008, Eimil-Ortiz et al. 2008
•	Atypical attack duration and frequency [Todo & Inoya 1991, Favier et al. 2007a case 2, Eimil-Ortiz et al. 2008
•	Atypical attack duration and abnormal findings on neurologic examination [Mainardi et al. 2002, Razvi et al. 2006, Park et al. 2006, Massie et al. 2006, Favier et al. 2007a case 2, Rigamonti et al. 2007 case 1, Hardmeier et al. 2007, Tobin & Flitman 2008, Liu & Su 2009
•	Did not meet the criterion of five attacks [Todo & Inoya 1991
•	Continuous headache or a background headache [Hannerz 1989, West & Todman 1991, Todo & Inoya 1991, Taub et al. 1995, Frigerio et al. 2003, Favier et al. 2007a case 3, Hardmeier et al. 2007, Kim et al. 2008, Harley & Ahmed 2008
• Atypical symptoms:	
	Impotence [Tfelt-Hansen et al. 1982
	Symptoms of acromegaly [Milos et al. 1996
	Episodes of altered consciousness [Munoz et al. 1996 case 2, Favier et al. 2007a: case 2]
	Headache triggered by sitting or standing [Piovesan et al. 2001
	and purulent nasal discharge [Scorticati et al. 2002
	Acute weakness in the upper extremity [Liu & Su 2009
• Physical abnormalities on clinical examination:	
	Testicular atrophy [Tfelt-Hansen et al. 1982
	Ophthalmoplegia [Hannerz 1989, Todo & Inoya 1991, Taub et al. 1995, Mainardi et al. 2002, Frigerio et al. 2003, Hannerz et al. 2005, Razvi et al. 2006, Park et al. 2006, Favier et al. 2007a: case 2: Favier et al. 2007b, Rigamonti et al. 2007 case 1 and 2, Hardmeier et al. 2007, Straube et al. 2007 case 2, Valença et al. 2007 case 1 and 2, Godeiro-Junior et al. 2008, Tobin & Flitman 2008, Ashkenazi & Brown 2008
	Optic atrophy [Tfelt-Hansen et al. 1982
	Papilloedema [Park et al. 2006
	Bitemporal hemianopia [Favier et al. 2007b
	Adie syndrome [Favier et al. 2007a: case 2]
	Persistent partial or complete Horner syndrome [Mainardi et al. 2002, Frigerio et al. 2003, Hannerz et al. 2005, Razvi et al. 2006 Rigamonti et al. 2007 case 1 and 2, Hardmeier et al. 2007, Straube et al. 2007 case 2, Godeiro-Junior et al. 2008, Tobin & Flitman 2008
	Signs of acromegaly [Milos et al. 1996
	Absent radial pulse [Piovesan et al. 2001
	Trigeminal distribution numbness [Massie et al. 2006, Gentile et al. 2007, Ashkenazi & Brown 2008, Alty et al. 2008, Liu & Su 2009
	Swelling of the eye [Favier et al. 2007a: case 3, Ashkenazi & Brown 2008, Kim et al. 2008
	Absent nasal tickle reflex [Massie et al. 2006
	Purulent nasal discharge [Scorticati et al. 2002

*References.

Fourteen patients had episodic CH (4, 12, 16, 17: case 2; 18, 19, 21, 30, 31, 34: case 2; 49, 51, 52, 54), 14 patients had chronic CH (5, 7: case 3; 10, 11, 15, 20, 23, 27, 32, 33, 34: cases 3 and 4; 35, 61). In the remaining 35 patients it was not possible to classify CH mainly because the patients were diagnosed and treated within 1 year.

Thirty patients of the 63 patients had a disappearance of the headache after medical therapy aimed at the structural lesion. Different treatments were used as antibiotics (sinusitis), corticosteroids (multiple sclerosis, orbital pseudotumor, sarcoidosis), anticoagulation/antiplatelet treatment (dissection, thrombosis), dopamine agonist treatment (prolactinoma), radiotherapy/chemotherapy (nasopharynx carcinoma).

Fifty-three of all patients had an ipsilateral lesion. Nine patients had a bilateral lesion (with unilateral attacks) and in 1 patient the lesion was central (Arnold-Chiari malformation with syringomyelia).

## Discussion

The aim of this review was to identify clinical signs and symptoms predictive of underlying abnormalities and thus better the diagnostic assessments of CH. Sixty-three patients with symptomatic CH were identified. It is difficult to draw definitive conclusions from this retrospective review of case reports especially considering the size of the material. The fact that the patients improved or recovered could be due to e.g. a strong placebo effect, spontaneous fluctuations in the severity of the disease or to a temporary or permanent resolution in an episodic disease (Mainardi et al. 2010; Wilbrink et al. 2009). Given the prevalence the number of reported cases of symptomatic CH in the literature is low. This suggests that symptomatic CHs are rare.

An underreporting in the literature of the actual number of cases of symptomatic CH is however likely. A significant portion of the reported patients met the criteria for CH according to ICHD-2 (Headache Subcommittee of the International Headache Society 2004). It may indicate that the actual numbers of patients with CH who have an underlying lesion are larger than previously assumed. Current criteria stipulate that CHs may only be diagnosed when an underlying disease has been excluded as the cause of the headache. However, is not defined in the criteria when such an investigation should be performed. A lack of neuroimaging in patients with CH could explain the low number of reported cases of symptomatic CHs. Given the number of cases reporting an improvement/disappearance of CH after an intervention directed at a supposedly underlying lesion it is likely that CHs, at least in some cases, are secondary to a treatable lesion (Wilbrink et al. 2009).

Some articles recommend neuroimaging in all patients with CH (Favier et al. 2007a; Mainardi et al. 2010; Wilbrink et al. 2009). The reason for this is that large structural lesions may present as typical episodic CH and also respond to established therapy. With this approach the clinician will most likely identify a significant number of incidental lesions, e.g. incidental pituitary microadenomas which could then be erroneously considered to be the cause of the CH (Lambru & Matharu 2012). Other authors suggest that symptomatology and objective signs (Cittadini & Matharu 2009; Favier et al. 2008; Lambru & Matharu 2012) and treatment results (Cittadini & Matharu 2009; Lambru & Matharu 2012) should determine whether further investigations are indicated.

CH onset usually occurs between the third and fifth decade (Nesbitt & Goadsby 2012). The peak incidence for both sexes occurs between age 20–29 (Ekbom et al. 2002). Late onset of CH should in itself lead to increased attention (Mainardi et al. 2010). In this review of symptomatic cases, the mean age of symptom onset was 40 years, which supports the opinion that late onset CH should prompt careful evaluation.

There is an interval between clinical onset and diagnosis in symptomatic CH. In this review, the average time passed between symptom onset and correct diagnosis proved to be 4 years. Therefore, the correct diagnosis in symptomatic CH, which so closely mimics CH, presents a clinical challenge. A delay in diagnosis of symptomatic CH might mean a great risk for the patient. Patients with a presumed diagnosis of CH should therefore be accurately evaluated to rule out symptomatic CH.

The ratio between male and female is 4.4:1 in clinical populations of CH. The ratio has decreased in the last decades, possibly due to increased awareness that also females can suffer from CH (Olesen et al. 2006). Furthermore, it has been suggested that this change may be due to alterations in lifestyle of both genders over the past few decades. A study from 2002 reported that the overall male-to-female ratio in the sample was 2.5:1 (Bahra et al. 2002). In this study, gender ratio was 3.2:1, which is in line with most of the patient sample studies. However, male preponderance is not expected in symptomatic CH. The underlying lesions as a whole are not considered to be gender related or genetically determined. The observed male preponderance in symptomatic CHs needs to be elucidated in further studies.

In many articles there was only limited information about the response to specific headache therapy. The response of the headache to sumatriptan and oxygen and other typical CH medications does not exclude a secondary form. The headache attacks may be clinically indistinguishable from the primary form (Favier et al. 2007a). Symptomatic CHs responsive to this therapy have been described (Ad Hoc. Committee on Classification of Headache of the National Institutes of Health 1962; Cremer et al. 1995).

Of the 63 cases reported in this review, 30 had a disappearance of the headache after medical therapy aimed at the structural lesion. It is important to stress that because of the temporal pattern of CH, the disappearance of the headache attacks might be attributed to the remission of the active phase in the episodic form, even in the cases with a documented pathology. A spontaneous remission of an episodic CH could be misinterpreted as being an effect of therapy aimed at the structural lesion. All the patients in the review were reported as showing resolution of the headache syndrome after treatment of the underlying pathology though the follow-up period was not stated in all cases and was fairly short in other cases. However, many patients have remained free of CH attacks at the follow-up after many years. This fact points to an association between the intervention and the resolution of the headache.

The exact pathophysiology in these cases of symptomatic CH is unknown. A structural lesion may cause autonomic imbalance, resulting in periodic fluctuations in the activity of the autonomic nervous system, ultimately leading to an attack-wise presentation of the symptoms (Wilbrink et al. 2009). Differences in the individual threshold for triggering the parasympathetic trigeminal reflexes may also play a role (Straube et al. 2007). The pain mechanism in secondary CH seems ascribable to irritation of pain-sensitive structures and activation of trigeminal nerve endings (Leone & Bussone 2009).

The low prevalence of glial tumours associated with symptomatic CHs is remarkable. Only one report has been published showing an association between CH and glioma (Edvardsson & Persson 2012). A possible mechanism could be the infiltrating nature of a glial tumour which lowers its potential to act on structures triggering symptomatic CH (Mainardi et al. 2010).

A striking finding in this appraisal was that a significant proportion of CH cases were secondary to diseases of the pituitary gland or pituitary region. Of the 63 cases, 14 (22%) were diseases in this region, with 10 cases of pituitary tumours, 1 case report of a granulomatous hypophysitis, 1 case report of an arachnoid cyst, 1 case report of a hypothalamic sarcoidosis and 1 case report of a meningioma. In all 10 cases of pituitary tumours the headache resolved completely after treatment. In 7 cases there was medical treatment (dopamine agonist treatment). Two cases had surgery. One case had surgery and radiotherapy.

Other articles have reported similar findings (Favier et al. 2007a; Favier et al. 2008; Wilbrink et al. 2009; Cittadini & Matharu 2009; Mainardi et al. 2010; Lambru & Matharu 2012). Headache is a common symptom of pituitary disease. In a large observational study of pituitary tumours and headache (84 patients), 4% had CH. Functioning rather than nonfunctioning adenomas were more likely to be associated with CH. However, the study was conducted in a tertiary referral neurosurgical centre and, therefore, does not give a meaningful indication of the prevalence of these headaches in patients with pituitary disorders (Levy et al. 2005; Cittadini & Matharu 2009; Lambru & Matharu 2012). It is still unclear whether the prevalence of pituitary tumours is higher in CH patients as clarifying studies are lacking. Approximately one in 10 of the general population has an incidental pituitary microadenoma (<1 cm diameter) on routine MRI, and up to one in 500 will have a macroadenoma (Ezzat et al. 2004; Cittadini & Matharu 2009; Lambru & Matharu 2012). Thus, it is not uncommon that MRI reveals a pituitary lesion in headache patients. The diagnostic workup of CH remains unclear. In view of this, it is reasonable to recommend that all patients with CH should be carefully assessed for symptoms/objective signs of pituitary gland/pituitary region disease and further investigations should be undertaken when needed.

Another notable finding was that a proportion of CH cases were associated with arterial dissection. Pain in internal carotid artery dissection is postulated to be caused by stimulation of the trigeminovascular system and it can mimic different primary headaches, including CH (Biousse et al. 1994). Examination during both primary CH and internal carotid artery dissection may demonstrate a Horner’s syndrome. Persistent ptosis and miosis between headaches are widely accepted as features of primary CH. Duration of headache more than three hours, absence of daily periodicity, neck pain, and no worsening from alcohol should increase the degree of suspicion of dissection (Razvi et al. 2006; Godeiro-Junior et al. 2008).

A non-typical presentation was seen in 52% of the cases. Some of the cases developed objective signs and atypical symptomatology during the course of the disease. Thus, the presence of atypical symptomatology such as abnormal attack duration (17 patients), development of continuous headache or background headache (9 patients), other abnormal symptoms of primary headache (8 patients) and abnormal clinical examination (29 patients) in the initial stage or during the course of the disease should prompt further investigations and should be considered as warning signs of secondary headaches.

It is noteworthy that 48% of the cases had a typical CH according to the criteria without evidence of any underlying pathology in the initial stage. Furthermore, in many cases, it took several years before the underlying cause was identified. This was in many cases due to the fact that the patients initially had a typical CH and later developed objective signs/symptoms during the course of the disease which prompted further investigations (Cittadini & Matharu 2009). In 3 cases, the initial evaluation with computer tomography was normal but a subsequent MRI revealed the underlying pathology. What implications does this have for the management of patients with cluster headache i.e. what investigations should be carried out and which patients should be assessed? The answer to that question will have to wait until a large prospective study is carried out. On the basis of available information, it is recommended that MRI including magnetic resonance angiography (MRA) should be carried out in all patients with atypical symptoms, abnormal clinical examination, late onset of symptoms and in patients with therapy resistance to appropriate treatments.

In patients with typical CH with respect to age at onset, symptoms, clinical examination, and response to therapy the question of investigation is more in dispute. The decision to perform neuroimaging in these patients remains a matter of medical art. If no further investigations are undertaken, the patient may be followed up in order to detect any abnormalities later during the course of the disease.

## Conclusions

CH is a primary headache. The great majority of cases are primary. In the initial assessment, medical history and clinical examination are of vital importance and can point to secondary causes of the headache. In patients with typical CH with respect to age at onset, symptoms, clinical examination, and response to therapy the patients may be followed up in order to detect any abnormalities during the course of the disease. However, some articles recommend that all patients with CH should be investigated with MRI. A significant portion of the cases in the review were secondary to diseases of the pituitary/pituitary region and arterial dissection. All patients with CH should be especially assessed for the possibility of pituitary region disease/arterial dissection. MRI including MRA should be undertaken in patients with atypical symptoms, abnormal clinical examination (including Horner's syndrome), late onset of symptoms and in patients with therapy resistance to appropriate treatments. Prospective studies are needed to identify the prevalence of symptomatic CHs.
